# Low anemia but high dyslipidemia prevalence in Brazilian schoolchildren: a nutritional transition profile

**DOI:** 10.1038/s41430-026-01736-z

**Published:** 2026-04-04

**Authors:** Ana Carolina Marques Ciceri, Syang Ândrea de Oliveira, Luisa Buhse Pasqualoto, Michelle Kaefer, Laura Eduarda de Oliveira, Natália Flores Jacobi, Laura Bonai Casal, Nicole Carvalho Hoppe, Ighor Seiji Okumura Tioda, José Antonio Mainardi de Carvalho, Clóvis Paniz

**Affiliations:** 1 Postgraduate Program in Pharmaceutical Sciences, Center of Health Sciences, Federal University of Santa Maria, Santa Maria, Brazil; 2 Applied Clinical Analysis Research Laboratory (LAPACA), Department of Clinical and Toxicologic Analysis, Center of Health Sciences, Federal University of Santa Maria, Santa Maria, Brazil; 3 University Hospital of Santa Maria, Federal University of Santa Maria, Santa Maria, Brazil

**Keywords:** Biomarkers, Risk factors, Pathogenesis, Biochemistry, Medical research

## Abstract

**Background/Objectives:**

Brazil is undergoing a rapid nutritional transition, yet data on anemia and dyslipidemia in early school-aged children remain scarce. This study aimed to determine the prevalence of anemia and dyslipidemia and to investigate the relationship between anemia, iron status markers, inflammation, lipid profiles, and nutritional status in schoolchildren from southern Brazil.

**Subjects/Methods:**

This cross-sectional study enrolled 204 children aged 5–8 years attending public municipal schools. Data collection included anthropometry, sociodemographic and dietary questionnaires, and blood analysis for hemoglobin, ferritin, transferrin saturation, reticulocyte hemoglobin equivalent (Ret-He), C-reactive protein (CRP), and lipid profile, using standard pediatric cut-offs.

**Results:**

Anemia prevalence was low (4.9%; 95% CI: 2.7–8.8%), whereas dyslipidemia prevalence was high, including hypertriglyceridemia (26.8%; 95% CI: 21.2–33.3%) and elevated total cholesterol (31.9%; 95% CI: 25.9–38.6%). Ret-He was significantly lower in anemic children (29.9 vs. 32.7 pg; p < 0.001) and correlated with hemoglobin (Rho=0.335, p < 0.001) and serum iron (Rho=0.280, p < 0.001). CRP was the strongest independent predictor of Ret-He (β = -0.345, p < 0.001), independent of nutritional status and conventional iron markers. Ret-He values did not differ across nutritional status categories, whereas inflammatory and lipid parameters varied according to adiposity. Meanwhile, soda intake was associated with higher triglyceride levels.

**Conclusions:**

These findings indicate a nutritional transition profile characterized by low anemia but high dyslipidemia prevalence in children aged 5–8 years. Ret-He reflects functional iron availability and is strongly influenced by systemic inflammation, independently of nutritional status, supporting its use as a complementary marker in the assessment of anemia in pediatric populations.

## Introduction

Anemia and dyslipidemia are major public health concerns in children, with profound implications for growth, development, and long-term cardiometabolic health. Anemia affects about 600 million children globally, nearly half of them school-age, with the highest burden in low- and middle-income countries due to socioeconomic disparities, nutritional deficiencies, and poor maternal health [1–3].

The World Health Organization (WHO) defines anemia as a hemoglobin concentration < 11.5 g/dL in children aged 5–12 years [4], with causes including impaired erythropoiesis, increased erythrocyte destruction, or blood losses [5]. Iron deficiency remains the leading cause, affecting up to 50% of children, especially in impoverished regions [6–8]. Its consequences include impaired cognitive and motor development, reduced academic performance, and possible irreversible neurological damage, even after supplementation [7, 9]. Besides inadequate intake, parasitic infections, chronic inflammation, and poor access to nutrient-rich foods also affect iron status [10, 11].

Concurrently, dyslipidemia, characterized by abnormal levels of total cholesterol, low-density lipoprotein cholesterol (LDL-C), high-density lipoprotein cholesterol (HDL-C), and triglycerides, is increasingly observed in pediatric populations, partly due to obesity and diet rich in energy-dense nutrient poor foods [12, 13]. Childhood dyslipidemia predicts early atherosclerosis, with subclinical vascular changes beginning in youth and persisting into adulthood, raising lifetime cardiovascular risk [12, 14].

Evidence suggests a link between iron metabolism and lipid homeostasis. Some studies report inverse associations between serum transferrin or soluble transferrin receptor levels and dyslipidemia risk in children [10]. The “iron hypothesis” proposes that iron overload may promote oxidative stress and lipid peroxidation, whereas iron deficiency may disrupt metabolic pathways regulating lipids. However, data on the co-occurrence of anemia and dyslipidemia in children remain scarce, especially in Latin America.

Given the simultaneous burden and possible biological interplay, particularly in socioeconomically vulnerable settings, investigating prevalence and determinants of anemia and dyslipidemia is crucial. Therefore, the objective of this study was to assess the prevalence of anemia and dyslipidemia and to evaluate associated hematologic parameters, iron status, inflammatory and lipid profiles among children aged 5 to 8 years attending public schools from southern Brazil, including comparisons according to nutritional status.

## Material And Methods

### Participants

This cross-sectional study enrolled 216 children aged 5 to 8 years from public elementary schools in Santa Maria, Rio Grande do Sul, Brazil. Data collection occurred between August 2022 and May 2025. Schools were selected if they primarily served children from socially vulnerable areas and agreed to participate after formal invitation. All first-grade students were invited, and inclusion and exclusion criteria were defined a priori. Inclusion required written parental consent and child assent. Exclusion criteria were missing blood count data or age outside 5–8 years. After exclusions, 204 students remained. As this was an exploratory, observational cross-sectional study based on a school-based population, a formal a priori sample size calculation was not performed. All eligible children from the selected schools during the study period were invited to participate, and the final sample size was determined by feasibility. Given the observational nature of the study, no randomization procedures were applied, and investigators were not blinded to group allocation. The study followed ethical standards and was approved by the Ethics Committee of the Federal University of Santa Maria (CAEE 29792120.9.0000.5346).

### Sample collection and analysis

Blood samples were collected in the morning after an 8-hour fasting period. Venipuncture was performed using Vacutainer® vacuum tubes (BD Diagnostics, Plymouth, UK). EDTA tubes were used for hematology and additive-free tubes for serum. Hematological analyses were conducted using the Sysmex XN-1000 automated hematology analyzer (Sysmex, Kobe, Japan). The complete blood count included red blood cell (RBC) count, hemoglobin concentration (HGB), hematocrit (HCT), mean corpuscular volume (MCV), mean corpuscular hemoglobin (MCH), mean corpuscular hemoglobin concentration (MCHC), red blood cell distribution width (RDW), reticulocyte count (RET), and reticulocyte hemoglobin equivalent (Ret-He).

Biochemical assays were analyzed on the Dimension® EXL™ 200 (Siemens, New York, USA), measuring serum iron, ferritin, total iron binding capacity (TIBC), C-reactive protein (CRP), total cholesterol, HDL-C and triglycerides. Transferrin saturation (TSAT) was calculated using the formula: (serum iron/ TIBC) x 100. Non-HDL cholesterol fraction was derived by subtracting HDL-C from total cholesterol. LDL-C values were estimated using the Friedewald formula, as recommended by Brazilian Society of Cardiology [15].

### Data collection

On the same day, weight and height were measured with calibrated equipment. Parents or guardians completed a questionnaire covering sociodemographics, breastfeeding duration, birth weight, siblings, chronic diseases, medication use, and frequency of key regional foods. Dietary frequency options were: never, monthly, weekly, 2–3 times/week, or daily. BMI was calculated (kg/m²), and z-scores derived using WHO growth standards (2007) [16]: [(BMI /M)^L^ – 1] / L x S, where L, M, and S are curve parameters.

### Cut-off points

Anemia was defined as hemoglobin <11.5 g/dL [4]; iron deficiency as ferritin <15 µg/L [17]. Dyslipidemia cut-offs for 5–9 years were: total cholesterol >170 mg/dL, LDL-C > 110 mg/dL, HDL-C < 45 mg/dL, non-HDL > 120 mg/dL, and triglycerides >75 mg/dL, as recommended by Brazilian and international guidelines [15].

### Statistical analysis

Analyses were conducted with SPSS v27.0 (IBM, USA) and GraphPad Prism v9.0 (USA). Data normality was assessed with the Kolmogorov-Smirnov test. As distributions were non-normal, results were expressed as medians (IQR). Group comparisons were performed using the Mann–Whitney U test for continuous variables. For comparisons across three nutritional status categories, the Kruskal–Wallis test followed by Dunn’s post-hoc test was applied when appropriate. For categorical data, the Chi-square test or Fisher’s Exact test was used. Spearman’s rank correlation was applied to assess associations. To identify determinants of Ret-He, multiple linear regression was performed including ferritin, TSAT, CRP, BMI-for-age z-score, age, sex, family income, maternal education, and soda intake. CRP was log-transformed to reduce skewness. Multicollinearity among predictors was assessed using Variance Inflation Factors (VIF). Additionally, a sensitivity analysis was performed by refitting the regression model excluding BMI-for-age z-score to evaluate whether nutritional status influenced the association between inflammation and Ret-He. Given the non-normal distribution of the data, non-parametric statistical tests were used; therefore, the assumption of homogeneity of variances between groups was not required. Significance was set at *p* < 0.05.

## Results

The study population comprised 204 children aged 5–8 years, of both sexes, enrolled in public schools in a city located in the southern region of Brazil. The median age of the population was 6 years, with no differences between boys and girls. Additional relevant characteristics, including body mass index (BMI), type of birth, birth weight, duration of breastfeeding, and others, are demonstrated in Table 1.

**Table 1:** Sociodemographic and anthropometric characteristics of schoolchildren aged 5–8 years from public schools in southern Brazil.

	All subjects (n = 204)	Girls (n = 97)	Boys (n = 107)	*p*
Age (y)	6 (6–7)	7 (6–7)	6 (6–7)	0.255
BMI (Kg/m²)	15.7 (14.5–17.9)	15.4 (14.2–18.6)	16.1 (14.8–17.9)	0.102
z-scores				0.453*
Severe thinness (< -3 SD)	3 (1.5)	1 (1.0)	2 (1.9)	
Thinness (≥ -3 to < -2 SD)	4 (2.0)	2 (2.1)	2 (1.9)	
Healthy (≥ -2 to ≤ +1 SD)	134 (65.7)	68 (70.1)	66 (61.7)	
Overweight (> +1 to ≤ +2 SD)	30 (14.7)	12 (12.4)	18 (16.8)	
Obesity (> +2 to ≤ +3 SD)	18 (8.8)	10 (10.3)	8 (7.5)	
Severe obesity (< +3 SD)	15 (7.4)	4 (4.1)	11 (10.3)	
Self-reported skin color				0.186
White	137 (67.2)	59 (60.8)	78 (72.9)	
Black	21 (10.3)	12 (12.4)	9 (8.4)	
Mulatto	46 (22.5)	26 (26.8)	20 (18.7)	
Birth weight > 2.5 Kg	172 (84.3)	82 (84.5)	90 (84.1)	0.934
Vaginal delivery	105 (51.5)	45 (46.4)	60 (56.1)	0.167
Breastfeeding				0.628
Did not breastfeed	22 (10.8)	10 (10.3)	12 (11.2)	
≤ 6 mo	43 (21.1)	18 (18.6)	25 (23.4)	
Between 6 mo - 1 y	25 (12.3)	9 (9.3)	16 (15.0)	
Between 1 y - 2 y	44 (21.6)	22 (22.7)	22 (20.6)	
Between 2 y - 3 y	39 (19.1)	22 (22.7)	17 (15.9)	
> 3 y	31 (15.2)	16 (16.5)	15 (14.0)	
Number of siblings				0.604
0	28 (13.7)	14 (14.4)	14 (13.1)	
1	75 (36.8)	38 (39.2)	37 (34.6)	
2 – 4	79 (38.7)	33 (34.0)	46 (43.0)	
> 4	22 (10.8)	12 (12.4)	10 (9.3)	
Birth order	2 (1–3)	2 (1–3)	2 (1–3)	0.475
Mother’s educational level				0.203*
No formal education ( < 1 y)	2 (1.0)	0 (0.0)	2 (1.9)	
Elementary (1–8 y)	79 (38.7)	39 (40.2)	40 (37.4)	
Secondary (9–11 y)	107 (52.5)	53 (54.6)	54 (50.5)	
Higher ( > 11 y)	16 (7.8)	5 (5.2)	11 (10.3)	
Familiar monthly income**				0.447*
< 1 minimum wage	47 (23.0)	23 (23.7)	24 (22.4)	
1 to 2 minimum wage	123 (60.3)	60 (61.9)	63 (58.9)	
2.1 to 5 minimum wage	28 (13.7)	13 (13.4)	15 (14.0)	
> 5 minimum wage	6 (2.9)	1 (1.0)	5 (4.7)	

^*^Likelihood ratio.

** Value of minimum wage in Brazil in the year of 2025 is R$ 1,518.00, approximately $ 285 dollars. BMI: body mass index. Z-scores: the z-score was calculated based on BMI and the growth reference tables with curves for boys and girls aged 5–19 y, from the WHO. Continuous variables are shown as median and interquartile ranges values (in parentheses). The groups were compared by Mann-Whitney test. Categorical variables are shown as number of subjects and percentage (in parentheses). The groups were compared by Chi-square test.

The prevalence of anemia was low in this population (4.9%; 95% CI: 2.7–8.8%). In contrast, a high frequency of dyslipidemia was observed, with elevated total cholesterol in 31.9% of the children (95% CI: 25.9–38.6%) and hypertriglyceridemia in 26.8% (95% CI: 21.2–33.3%). In addition, a substantial proportion of children, 24.5% presented low HDL-C levels (95% CI: 19.1–30.8%), further characterizing an unfavorable lipid profile (Table 2). Furthermore, hematological and biochemical parameters were largely comparable between boys and girls. However, notable differences were observed for RBC, MCV, MCH, RDW, and TIBC. An overview of the parameters and values is presented in Table 2.

**Table 2:** Hematological and biochemical parameters of the study population stratified by sex.

	All n = 204	Girls n = 97	Boys n = 107	*p*
RBC (x10^6^/µL)	4.7 (4.5–4.9)	4.6 (4.4–4.8)	4.8 (4.6–5.0)	0.009
HGB (g/dL)	12.7 (12.1–13.1)	12.5 (12.1–13.0)	12.8 (12.3–13.1)	0.146
HCT (%)	37.3 (35.7–38.7)	37.1 (35.6–38.9)	37.5 (35.8–38.7)	0.508
MCV (fL)	79.4 (76.9–81.8)	80.5 (78.0–82.3)	78.7 (76.3–80.5)	< 0.001
MCH (pg)	27.1 (26.1–27.7)	27.3 (26.4–27.9)	26.9 (26.0–27.5)	0.028
MCHC (g/dL)	33.9 (33.3–34.6)	33.8 (33.3–34.4)	34.1 (33.3–34.8)	0.098
RDW (%)	12.9 (12.5–13.4)	12.8 (12.4–13.2)	13.2 (12.6–13.5)	< 0.001
Reticulocyte* (x10^3^/µL)	58.5 (46.8–68.4)	60.1 (47.8–68.6)	57.1 (44.3–68.2)	0.457
Ret-He* (pg)	32.7 (31.4–33.8)	32.9 (31.8–34.0)	32.5 (31.2–33.6)	0.129
Iron (µg/dL)	77 (56–101)	74 (59–99)	78 (53–101)	0.981
Ferritin (µg/dL)	54 (40–72)	52 (40–70)	55 (37–73)	0.650
TIBC** (µg/dL)	260 (221–293)	245 (211–276)	274 (239–303)	< 0.001
TSAT** (%)	29.7 (20.5–42.0)	30.4 (22.5–45.3)	28.3 (19.5–38.6)	0.241
Total Cholesterol (mg/dL)	158 (139–178)	153 (140–174)	163 (139–181)	0.521
LDL-C (mg/dL)	91 (76–107)	88 (77–107)	95 (76–107)	0.470
HDL-C (mg/dL)	52 (45–60)	50 (43–59)	53 (45–62)	0.174
Non-HDL-C (mg/dL)	105 (89–121)	103 (90–122)	108 (88–121)	0.938
Triglycerides (mg/dL)	57 (45–79)	57 (47–81)	56 (44–77)	0.373
CRP (mg/dL)	0.18 (0.08-0.58)	0.18 (0.09-0.58)	0.17 (0.06-0.58)	0.870
HGB < 11.5 g/dL	10 (4.9)	4 (4.1)	6 (5.6)	0,751*
Ferritin <15 µg/dL	2 (1.0)	0 (0.0)	2 (1.9)	0,499*
Total Cholesterol >170 mg/dL	65 (31.9)	28 (13.7)	37 (18.1)	0,382**
HDL-Cholesterol <45 mg/dL	50 (24,5)	26 (26,8)	24 (22,4)	0,468**
Triglycerides >75 mg/dL	55 (26.8)	27 (27.6)	28 (26.2)	0,789**

^*^n = 194 (91 girls and 103 boys);

**n = 192 (94 girls and 98 boys); *RBC* red blood count, *HGB* hemoglobin concentration, *HCT* hematocrit, *MCV* mean corpuscular volume, *MCH* mean corpuscular hemoglobin, *MCHC* mean corpuscular hemoglobin concentration, *RDW* red cell distribution width, *RET* reticulocytes, *Ret-He* reticulocyte hemoglobin equivalent, *TIBC* total iron binding capacity, *LDL-C* low-density lipoprotein cholesterol, *HDL-C* high-density lipoprotein cholesterol, *Non-HDL-C* non-HDL cholesterol, CRP C-reactive protein. * Fisher’s exact Test; ** Pearson Chi-square Test.

Although the frequency of anemia was low, and the presence of anemia does not necessarily indicate iron deficiency anemia, lower Ret-He values were observed in anemic children [29.9 (26.9–32.3) pg] compared with non-anemic children [32.7 (31.7–33.8) pg] (*p* < 0.001) (Supplementary Table S1). When Ret-He was correlated with other variables related to anemia and iron status, weak positive correlations were observed with anemia (Rho = 0.240, *p* = 0.001, *n* = 194), hemoglobin concentration (Rho = 0.335, *p* < 0.001, *n* = 195), serum iron (Rho = 0.280, *p* < 0.001, *n* = 194), and transferrin saturation (Rho = 0.181, *p* = 0.014, *n* = 182). Ret-He was not correlated with ferritin concentrations or TIBC. CRP demonstrated negative correlation with Ret-He (Rho = −0.405, *p* < 0.001, *n* = 195), with TSAT (Rho = −0.300, *p* < 0.001, *n* = 193), and with serum iron (Rho = −0.407, *p* < 0.001, *n* = 205), indicating a potential role of inflammation in modulating functional iron availability.

Given this inflammatory context and the known links between inflammation, lipid metabolism, and nutritional status, we next examined the relationships between iron biomarkers and lipid parameters. Correlation between iron biomarkers and lipid parameters were weak or absent. Ferritin showed only a very weak negative correlation with HDL-C (rho = –0.170; *p* = 0.015), and Ret-He correlated weakly with total cholesterol (rho = 0.145; *p* = 0.044) and HDL-C (rho = 0.226; *p* = 0.002). No iron marker correlated with triglycerides or LDL. Full results are presented in Supplementary Table S2.

To further explore whether nutritional status could account for variability in inflammatory, iron, and lipid markers, participants were stratified into normal weight, overweight, and obese categories based on BMI-for-age z-scores. Children classified as thinness (*n* = 4) or severe thinness (*n* = 3) were excluded due to the small sample size. Biomarkers were compared across groups using the Kruskal–Wallis test followed by Dunn’s post hoc analysis. Ret-He values did not differ significantly between nutritional status categories (*p* = 0.364). In contrast, ferritin (*p* = 0.032), TIBC (*p* = 0.030), HDL-C (*p* = 0.019), total cholesterol (*p* = 0.001), triglycerides (*p* = 0.012), and CRP (*p* < 0.001) showed significant group differences. Post hoc analyses indicated that obese children presented higher ferritin and TIBC levels compared with overweight peers, while HDL-C was lower in overweight children. Total cholesterol, triglyceride, and CRP levels increased progressively from normal weight to obesity (Table 3).

**Table 3:** Biomarker comparison across nutritional status categories (normal weight, overweight, obesity).

Biomarker	Normal weight n = 134	Overweight n = 30	Obese n = 33	*p*
Ret-He (pg)	32.9 (31.6–33.9)	32.5 (31.7–33.4)	32.2 (30.8–32.8)	0.364
Ferritin (ng/mL)	51.6 (38.5–72.2)^a,b^	45.6 (38.8–62.4)^a^	67.1 (48.9–85.0)^b^	**0.032**
TSAT (%)	31.0 (21.6–43.7)	26.8 (15.8–41.2)	25.3 (19.3–34.1)	0.283
Serum iron (µg/dL)	78.5 (59.7–102.5)	66.0 (45.0–95.8)	73.0 (63.0–88.0)	0.238
TIBC (µg/dL)	252.5 (214.3–291.0)^a^	245.0 (221.8–291.0)^a,b^	276.0 (261.0–303.0)^b^	**0.030**
HDL-C (mg/dL)	52.5 (46.0–62.0)^a^	48.0 (40.7–53.0)^b^	55.0 (42.5–61.0)^a,b^	**0.019**
LDL-C (mg/dL)	92.0 (77.0–107.0)	85.0 (71.5–96.5)	98.0 (80.0–112.0)	0.065
Total cholesterol (mg/dL)	160.0 (137.0–181.0)^a^	142.0 (131.5–153.3)^b^	170.0 (152.0–194.5)^a^	**0.001**
Triglycerides (mg/dL)	53.0 (43.00–76.25)^a^	57.00 (43.50–71.25)^a,b^	73.00 (53.50–98.50)^b^	**0.012**
CRP (mg/dL)	0,13 (0,05-0,40)^a^	0,39 (0,10-1,03)^b^	0,30 (0,17-1,36)^b^	**<0,001**

*Ret-He* reticulocyte hemoglobin equivalent, *TSAT* Transferrin saturation, *TIBC* total iron binding capacity, *HDL-C* high-density lipoprotein cholesterol, *LDL-C* low-density lipoprotein cholesterol, *CRP* C-reactive protein. Within each row, different letters indicate statistically significant pairwise differences (Dunn’s test, *p* < 0.05).

Several factors may influence the etiology of dyslipidemia in children, including geographical location, local culture, lifestyle habits, socioeconomic conditions, and nutritional status.

No associations were observed between anemia and the consumption frequencies of the main food groups when comparing anemic and nonanemic children. Similarly, no associations were found between food group consumption and hypercholesterolemia after stratifying children into those with high vs. normal cholesterol concentrations. In contrast, higher consumption of soda was associated with greater serum triglycerides concentration (*p* = 0.002).

Family monthly income demonstrated weak positive correlation with higher meat intake (rho = 0.204, *p* = 0.003, *n* = 204), hemoglobin concentration (rho = 0.180, *p* = 0.010, *n* = 204), and serum glucose concentration (rho = 0.221, *p* = 0.002, *n* = 204); and a weak negative correlation with pasta intake (rho = −0.207, *p* = 0.003, *n* = 204). Except for meat intake, the consumption frequencies of other evaluated foods were not correlated with anemia or other laboratory parameters.

Furthermore, a weak positive correlation was observed between the mother’s educational level with the child’s hemoglobin concentration (rho = 0.178, *p* = 0.011, *n* = 204), serum total cholesterol (rho = 0.260, *p* < 0.001, *n* = 204) and non-HDL cholesterol (rho = 0.154, *p* = 0.027, *n* = 204). Type of delivery, birth weight, duration of breastfeeding, number of siblings, and birth order were not associated with the laboratory parameters evaluated.

To identify independent determinants of the Ret-He, a multiple linear regression analysis was performed. The final model included the following predictors: BMI-for-age z-score, log-transformed CRP, ferritin, age, sex, TSAT, family monthly income, and maternal educational level.

The overall regression model was statistically significant (F (8, 173) = 5.826, *p* < 0.001), explaining 21.2% of the total variance in Ret-He (R² = 0.212, Adjusted R² = 0.176). Analysis of the standardized coefficients (Beta) identified log-CRP as the strongest and only statistically significant predictor in the model, demonstrating a significant negative association with Ret-He (*β* = -0.345, *p* < 0.001). TSAT showed a positive association that approached, but did not reach, statistical significance (*β* = 0.125, *p* = 0.080). None of the other variables included in the model, such as ferritin (*β* = -0.074, *p* = 0.285), age (*β* = -0.095, *p* = 0.163), sex (*β* = 0.083, *p* = 0.229), BMI-for-age z-score (*β* = 0.047, *p* = 0.510), family income (*β* = 0.064, *p* = 0.393), and maternal education (*β* = 0.108, *p* = 0.147), were independent predictors of Ret-He. The sensitivity analysis excluding BMI-for-age z-score yielded results consistent with the main model, with log-CRP remaining the only independent predictor of Ret-He. Both models are now presented in Supplementary Tables S3 and S4.

Ret-He values did not differ significantly across nutritional status categories (normal weight, overweight, and obesity) (p = 0.364). Mean ranks showed a non-significant decreasing trend (97.5, 90.6, and 82.6, respectively), but not pairwise differences reached statistical significance. Full data are presented in Table 3.

## Discussion

This study provides one of the first Brazilian reports showing that, among public-school children, low anemia prevalence now coexists with an unfavorable lipid profile in many children. This pattern reflects the Brazilian nutritional transition, marked by advances in iron deficiency control and the emerging challenges of diet-related dyslipidemia.

The anemia prevalence observed (4.9%) was considerably lower than national and international reports (10% to 25%) among school-aged children, particularly in socioeconomically vulnerable groups [6, 7, 9]. Recent data suggest declining anemia rates in urban populations from Southern and Southeastern Brazil, suggesting a positive impact of public policies for the iron and folic acid fortification of flours, in addition to the promotion of breastfeeding [18, 19]. Despite the low prevalence, hematological variations by sex were noted, with boys showing lower MCV and MCH and higher RDW and TIBC, as previously described by Aldrimer et al. (2013) [20] and Zierk et al. (2015) [21]. Such differences likely represent physiological variations in iron metabolism but highlight the importance of follow-up in longitudinal studies.

Ret-He was included as a functional marker of iron availability for erythropoiesis, given its ability to reflect short-term changes in iron supply and its reported sensitivity to inflammation; therefore, it was analyzed alongside conventional iron markers [22]. In this study, lower Ret-He values were observed in anemic children compared with non-anemic peers, as demonstrated by the group comparisons reported in the Results section. These findings support the relevance of Ret-He as a functional marker of iron availability. Notably, in multivariable analysis, CRP emerged as the strongest independent predictor of Ret-He, supporting the role of low-grade inflammation in limiting functional iron availability even in the absence of overt anemia.

No consistent associations were observed between conventional iron markers (hemoglobin, ferritin, and transferrin saturation) and lipid parameters in the correlation analyses, indicating that anemia-related markers and dyslipidemia followed distinct distributions in this population. Together, these results suggest that anemia and dyslipidemia followed distinct distributions and represented separate, although potentially coexisting, nutritional challenges in this population.

Given the known association between obesity and systemic inflammation, multivariable models were tested with and without BMI z-score to evaluate potential collinearity between inflammation and adiposity. Variance inflation factors were consistently low for all variables (Supplementary Tables S3 and S4), and the association between CRP and Ret-He remained robust across models, indicating that the observed relationship was not driven by collinearity with nutritional status.

In contrast to the low prevalence of anemia, the frequency of hyperlipidemia was substantial in this population: approximately one-third of children exceeded total cholesterol thresholds, and 27% had hypertriglyceridemia. Additionally, nearly one quarter of the children exhibited low HDL-C levels, further characterizing an atherogenic lipid profile at an early age. Similar trends have been reported in Brazilian and international studies [15, 23–25]. A Chinese cohort (2004–2014) also documented rising triglyceride and cholesterol levels with reduced HDL-C among children and adolescents [26]. Such lipid alterations are known predictors of early atherosclerosis and long-term cardiovascular risk [27, 28]. Furthermore, these findings are consistent with the growing burden of childhood overweight and obesity, which have been associated with factors including sedentary behavior, dietary patterns, genetic predisposition, and social vulnerability, as demonstrated in previously described studies [29]. In Brazil, the prevalence of childhood overweight/obesity increased by 116% between 1992 and 2015 [30, 31], emphasizing the urgency of preventive action.

When analyses were stratified by nutritional status, progressive increases in ferritin, total cholesterol, triglycerides, glucose, and CRP were observed across categories, with the highest values in obese children. These findings reinforce the presence of early cardiometabolic risk associated with increasing adiposity [15, 27]. Elevated ferritin, often interpreted as iron overload, likely reflects inflammation in obesity rather than increased iron stores [32, 33]. This dual burden, even in low-income populations, highlights the coexistence of excess weight and metabolic alterations.

No significant associations emerged between iron markers (hemoglobin, ferritin, transferrin saturation) and lipid profile, suggesting anemia and dyslipidemia were largely independent conditions in this population. However, the consistent inverse relationship between CRP and Ret-He reinforces the role of inflammation as a key mediator linking adiposity and functional iron restriction. Elevated CRP in obese children supports the connection between adiposity, chronic inflammation, and early disturbances in iron metabolism. Interventions targeting childhood obesity may thus benefit both lipid regulation and iron metabolism.

Although socioeconomic and dietary variables were evaluated as contextual factors, their associations with laboratory markers were generally weak. Notably, soda intake was associated with higher triglyceride levels, consistent with other Brazilian studies [23], but did not influence iron markers or Ret-He. These findings suggest that, in this population, inflammation and nutritional status exert a stronger influence on iron availability and lipid profile than isolated dietary components.

These findings reinforce the need for integrated public health strategies. While Brazil has advanced in reducing anemia, measures targeting childhood dyslipidemia remain insufficient. Promoting minimally processed foods, breastfeeding, and physical activity are essential to reduce lifelong cardiometabolic risk.

Limitations include the cross-sectional design, lack of hormonal markers such as hepcidin, simplified dietary assessment, and restriction to a single city. Nonetheless, this study adds relevant data on anemia and dyslipidemia in Brazilian children and offers directions for future research. Multicenter longitudinal studies with novel biomarkers are needed to clarify interactions among iron metabolism, inflammation, and lipid alterations and to validate Ret-He reference intervals.

In conclusion, this study reveals a nutritional transition profile among Brazilian schoolchildren, characterized by a low prevalence of anemia alongside a high frequency of dyslipidemia. Although Ret-He values did not differ across nutritional status categories, this parameter proved to be a relevant functional marker of iron status and was independently associated with systemic inflammation, as reflected by CRP levels. The integrated assessment of hematological, inflammatory, and lipid biomarkers highlights the interplay between nutritional status and cardiometabolic risk early in life. These findings support the relevance of simple and widely accessible biomarkers as complementary tools for early risk stratification and public health surveillance in pediatric populations.

## Supplementary information

Table S1

Table S2

Table S3

Table S4

## Data Availability

The datasets used and/or analyzed during the current study are available from the corresponding author on reasonable request.
