# Supplementary material for: Low anemia but high dyslipidemia prevalence in Brazilian schoolchildren: a nutritional transition profile
Source: Eur J Clin Nutr. 2026 Apr 4;80(6):603–9. doi: 10.1038/s41430-026-01736-z (PMC13286996; doi:10.1038/s41430-026-01736-z)
Supplement: Supplementary file 1 — Table S1 [file 41430_2026_1736_MOESM1_ESM.docx]

**Table S1**. Comparison of reticulocyte hemoglobin (Ret-He) between anemic and non-anemic children

|  | Anemic  n= 10 | Non-anemic  n= 184 | Mann-Whitney U | Z | p |
| --- | --- | --- | --- | --- | --- |
| Ret-He (median, IQR), pg | 29.9 (26.9–32.3) | 32.7 (31.7–33.8) | 343 | -3,338 | <0,001 |
