# Supplementary material for: Low anemia but high dyslipidemia prevalence in Brazilian schoolchildren: a nutritional transition profile
Source: Eur J Clin Nutr. 2026 Apr 4;80(6):603–9. doi: 10.1038/s41430-026-01736-z (PMC13286996; doi:10.1038/s41430-026-01736-z)
Supplement: Supplementary file 2 — Table S2 [file 41430_2026_1736_MOESM2_ESM.docx]

**Table S2.** Spearman correlations between iron biomarkers and lipid parameters

| Iron parameter | Triglycerides (rho, p) | Total cholesterol (rho, p) | HDL-C (rho, p) | LDL-C (rho, p) |
| --- | --- | --- | --- | --- |
| Ferritin | 0.096 (0.173) | 0.097 (0.167) | **–0.170 (0.015)** | 0.124 (0.077) |
| TSAT | 0.085 (0.243) | –0.059 (0.420) | –0.031 (0.673) | –0.012 (0.866) |
| Serum iron | 0.077 (0.271) | 0.021 (0.771) | 0.068 (0.332) | 0.030 (0.666) |
| TIBC | –0.063 (0.383) | **0.202 (0.005)** | **0.247 (0.001)** | 0.098 (0.178) |
| Ret-He | –0.014 (0.850) | **0.145 (0.044)** | **0.226 (0.002)** | 0.052 (0.474) |

TSAT: Transferrin saturation; TIBC: Total iron bound capacity; Ret-He: reticulocyte hemoglobin equivalent; HDL-C: High-density lipoprotein cholesterol; LDL-C: Low-density lipoprotein cholesterol.
