# Supplementary material for: Low anemia but high dyslipidemia prevalence in Brazilian schoolchildren: a nutritional transition profile
Source: Eur J Clin Nutr. 2026 Apr 4;80(6):603–9. doi: 10.1038/s41430-026-01736-z (PMC13286996; doi:10.1038/s41430-026-01736-z)
Supplement: Supplementary file 3 — Table S3 [file 41430_2026_1736_MOESM3_ESM.docx]

**Table S3.** Multivariable regression models evaluating predictors of Ret-He, including collinearity diagnostics (VIF)

Model 1 — Full model (including BMI-for-age z-score)

Dependent variable: Reticulocyte hemoglobin (Ret-He)

| **Predictor** | **β (Unstandardized)** | **SE** | **β (Standardized)** | **95% CI for β** | **p-value** | **Tolerance** | **VIF** |
| --- | --- | --- | --- | --- | --- | --- | --- |
| **Intercept** | 32.673 | 1.459 | — | 29.792 to 35.553 | <0.001 | — | — |
| BMI-for-age z-score | 0.061 | 0.093 | 0.047 | –0.122 to 0.245 | 0.510 | 0.908 | **1.102** |
| **log-CRP** | **–0.405** | 0.089 | **–0.345** | –0.581 to –0.230 | **<0.001** | 0.796 | **1.256** |
| Ferritin | –0.004 | 0.004 | –0.074 | –0.011 to 0.003 | 0.285 | 0.944 | **1.059** |
| TSAT | 0.014 | 0.008 | 0.125 | –0.002 to 0.031 | 0.080 | 0.909 | **1.100** |
| Age | –0.301 | 0.215 | –0.095 | –0.725 to 0.123 | 0.163 | 0.990 | **1.011** |
| Sex | 0.323 | 0.268 | 0.083 | –0.205 to 0.852 | 0.229 | 0.973 | **1.027** |
| Family income | 0.124 | 0.145 | 0.064 | –0.162 to 0.410 | 0.393 | 0.820 | **1.219** |
| Maternal education | 0.334 | 0.229 | 0.108 | –0.119 to 0.786 | 0.147 | 0.828 | **1.208** |

BMI: Body mass index; CRP: C-reactive protein; TSAT: Transferrin saturation
