# Supplementary material for: Low anemia but high dyslipidemia prevalence in Brazilian schoolchildren: a nutritional transition profile
Source: Eur J Clin Nutr. 2026 Apr 4;80(6):603–9. doi: 10.1038/s41430-026-01736-z (PMC13286996; doi:10.1038/s41430-026-01736-z)
Supplement: Supplementary file 4 — Table S4 [file 41430_2026_1736_MOESM4_ESM.docx]

**Table S4.** Multivariable regression models evaluating predictors of Ret-He, including collinearity diagnostics (VIF)

Model 2 — Sensitivity analysis (BMI-for-age z-score removed)

Dependent variable: Reticulocyte hemoglobin (Ret-He)

| **Predictor** | **β (Unstandardized)** | **SE** | **β (Standardized)** | **95% CI for β** | **p-value** | **Tolerance** | **VIF** |
| --- | --- | --- | --- | --- | --- | --- | --- |
| **Intercept** | 32.743 | 1.453 | — | 29.875 to 35.611 | <0.001 | — | — |
| **log-CRP** | **–0.391** | 0.086 | **–0.333** | –0.561 to –0.221 | **<0.001** | 0.848 | **1.180** |
| Ferritin | –0.004 | 0.004 | –0.072 | –0.011 to 0.003 | 0.299 | 0.947 | **1.056** |
| TSAT | 0.015 | 0.008 | 0.125 | –0.002 to 0.031 | 0.078 | 0.910 | **1.099** |
| Age | –0.305 | 0.214 | –0.096 | –0.728 to 0.118 | 0.157 | 0.990 | **1.010** |
| Sex | 0.303 | 0.266 | 0.077 | –0.221 to 0.828 | 0.255 | 0.986 | **1.014** |
| Family income | 0.136 | 0.143 | 0.070 | –0.147 to 0.419 | 0.344 | 0.834 | **1.200** |
| Maternal education | 0.328 | 0.229 | 0.106 | –0.124 to 0.779 | 0.154 | 0.829 | **1.206** |

BMI: Body mass index; CRP: C-reactive protein; TSAT: Transferrin saturation
